# Statistical Mechanics Treatment of the Broadened Snoek Relaxation Peak in Ternary Niobium–Vanadium–Oxygen Alloys

**DOI:** 10.3390/ma11101948

**Published:** 2018-10-11

**Authors:** Jian Ren, Liming Yu, Yongchang Liu, Huijun Li, Zongqin Ma, Jiefeng Wu

**Affiliations:** 1 State Key Lab of Hydraulic Engineering Simulation and Safety, Tianjin Key Lab of Composite and Functional Materials, Tianjin University, Tianjin 300072, China; zzusimon@163.com (J.R.); ycliu@tju.edu.cn (Y.L.); huijun@uow.edu.au (H.L.); 2 Institute of Plasma Physics, Chinese Academy of Sciences, Hefei 230031, China; jfw@ipp.ac.cn

**Keywords:** statistical mechanics, relaxation, ternary bcc system

## Abstract

The Snoek relaxation profiles for ternary Niobium–Vanadium–Oxygen systems were analyzed by an embedded-cell model of statistical mechanics treatment. The relaxation characteristic and broadening mechanism were systematically discussed and some conflicting interpretations in the early research were clarified. The complicated Snoek spectrums of the Nb–V–O system can be resolved into a series of effective elementary Debye peaks, which result from the transitions of interstitial oxygen atoms between adjacent octahedral sites. The relaxation parameters of each elementary peak can be determined by element species and atomic arrangements within the corresponding embedded octahedron. The Snoek relaxation characteristic in Nb–V–O systems mainly depends on the sites distributions and the transitions status of the interstitial oxygen atoms, which are controlled by the site-dependence energies and the transition probabilities, respectively.

## 1. Introduction

Interstitial solute atoms present in free solid solution in bcc metals exhibit the well-known Snoek relaxation peaks [1]. The addition of substitutional solutes to these metals results in complex interactions among interstitial atoms, host- and substitutional-metal atoms. These interactions will markedly influence the anelastic relaxation processes. As a result, the Snoek relaxation profile in ternary bcc alloys usually exhibits complex characteristics such as broadening, asymmetry, or even new peak-formation, etc. [2,3]. On the other hand, a reasonable deconvolution result for the complex Snoek relaxation spectra based on the reasonable physical model could, in principle, provide a clearer picture of the concrete mechanisms of the relaxation process, the diffusion performances of the interstitials solutes, the complex interatomic interactions, and some detailed information of the local physical structure in ternary bcc alloys [4,5]. The group IV and V transition metals are ideally suited for this type of study due to their high solubility for interstitial solutes, notably oxygen and nitrogen [6]. Another important reason for highly researched interest in these systems is their excellent application potential as refractory metals [7]. The mechanical and thermal properties of these metals are sensitive to the interstitial solutes’ behavior, which can be suitably investigated by the point-defect relaxation profile [8]. In the past 50 years, many related investigations have been made, however, the explanations were quite rough, limited, and even contradictory [9].

**Figure 1:**
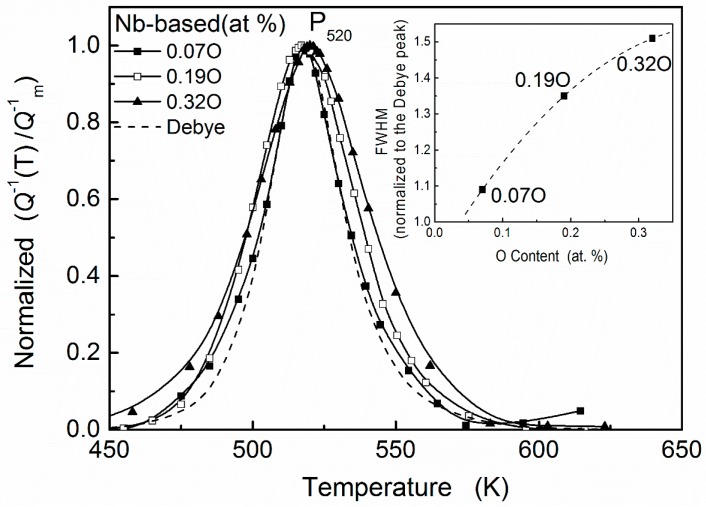
Normalized relaxation peaks for Nb–0.5V alloys with 0.07, 0.18, and 0.32 at. % O. Comparison with a theoretical Debye peak at 520 K with an activation energy determined by Marx–Wert equation.

In these research studies, ternary Nb–V–O systems attracted intensive attention [6,10,11,12,13,14]. The effects of a small addition of a substitutional solute to both niobium and vanadium on the oxygen Snoek peak of each metal were investigated in Carlson et al.’s work [6]. In Nb-based alloys, only one relaxation peak is present at about 520 K (inverted pendulum, 0.2–1.7 Hz) when the oxygen concentration is quite low. For convenience, we denote this experimental peak as P_II_ in the following section. Since the peak temperature is appreciably higher than that for the oxygen Snoek peak in pure niobium, P_II_ was attributed to a Snoek-type single relaxation process for an oxygen atom trapped by a substitutional vanadium atom (i.e., V–O peak). However, as shown in Figure 1, with increasing oxygen concentration, the peak width is broadened gradually. It indicates that peak P_II_ is not a simplex V–O interaction peak. Even with a highly diluted substitutional solute, the relaxation process of Nb-based Nb–V–O alloys has complex compositions.

When oxygen concentration is higher than vanadium content, i.e., $\frac{C_{O}}{C_{V}}$ > 1, P_II_ reaches a maximum height, and an additional peak (denoted P_I_) can be observed at about 400 K [6]. Carlson et al. explained this result suppositively that each vanadium solute atom in Nb-based alloys traps approximately one oxygen atom. As the oxygen concentration is increased, the trap sites become saturated and an unperturbed Nb–O Snoek peak, i.e., peak P_I_, appears. This is a rather speculative viewpoint since each substitutional atom in ternary bcc alloys has 6 nearest and 12 next nearest octahedral interstitial sites, which can be occupied by the interstitial atoms. Meanwhile, a controversial interpretation [15,16] has been developed that the interstitial solutes may interact with the matrix atoms in the form of pairs, triplets, and other small clusters, as has been deduced from elastic after-effect (EAE) and internal friction measurements.

**Figure 2:**
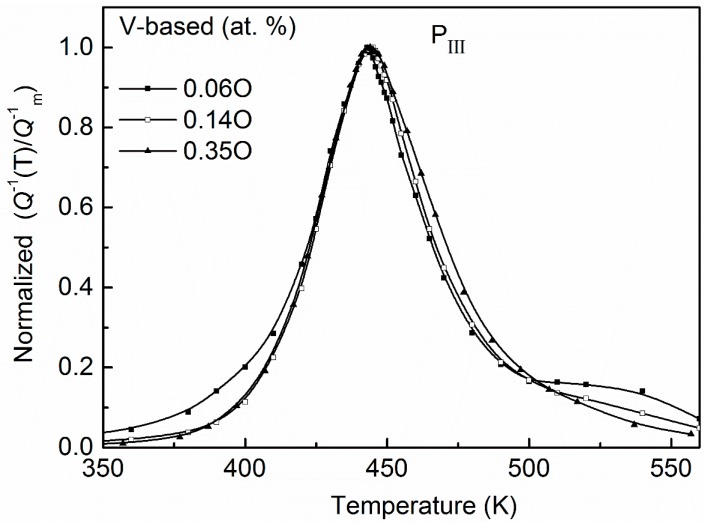
Normalized relaxation peaks for Nb–99.5V alloys with 0.06, 0.14, and 0.35 at. % O. No significant broadening effects appear with increasing of oxygen concentration.

On the other hand, the relaxation characteristic of the V-based Nb–V–O system was different from the case of Nb-based alloys. For example, in V-based Nb–99.5V (at. %) alloy, only one peak (denoted P_III_) occurs at about 450 K. The normalized relaxation peaks for Nb–99.5V (at. %) systems with different oxygen concentrations are shown in Figure 2. It can be seen that there are no significant broadening effects with the increasing of oxygen content. Since this peak temperature is close to that of the Snoek peak in vanadium as reported by various investigators, Carlson et al. interpreted it as an unperturbed V–O Snoek peak and as evidence that there is no trapping, but antitrapping, of oxygen by Nb in this V-rich alloy. However, this interpretation was controversial to the kinetic measured results of oxygen in Nb–V alloys by Park and Altstetter [17].

In summary, it is desirable to get a further clarification on the origin of the complex Snoek relaxation profile for ternary Nb–V–O systems. Recently, an extended statistical mechanical method [4,18], which is called embedded-cell method, has been proposed for directly analyzing the complicated Snoek relaxation peak in a ternary bcc system. Thus, it is now possible to reanalyze some of the results from these earlier investigations in the Nb–V–O systems in order to acquire new information which can help to understand reasons for the differing results and conflicting interpretations reported, and thus better describe the nature mechanism of the Snoek relaxation in these and other ternary bcc alloys. This is also the main aim of the present paper.

## 2. Method

In this method, interstitial sites of different geometries (octahedral, tetrahedral, etc.) in bcc crystals are statistically considered and further distinguished by the matrix atoms within their first shell of the neighbors [4]. Following these preconditions, the interstitial site occupancies, the spatial site configurations, and the transition probabilities of the interstitial solutes can be calculated in detail by using an embedded-cell model [4,19]. This information can be applied to predict all possible elementary processes of the Snoek relaxation in a ternary bcc system. Then, combining with a fitting method, the complicated Snoek peak can be resolved into several effective elementary Debye peaks, which correspond to the concrete relaxation mechanism. The detailed procedure of this method was described in the original Reference [4]. Here, we directly apply it to analyze the relaxation spectrums of the Nb–V–O system. Neutron diffraction and ion channeling studies [20,21] have shown that oxygen atoms occupy octahedral sites in Nb and V and it is assumed that they behave likewise in bcc Nb–V alloys under thermal equilibrium conditions. Thus, in this system, seven octahedral interstitial sites $I_{u}^{O}$ (*u* = 0, 1, 2, …, 6) can be distinguished depending on the number (*u*) of substitutional atoms within the first shell. Here and in the following section, the superscript ‘*O*’ and ‘*T*’ represent the octahedral and tetrahedral interstice, respectively.

### 2.1. Static Distribution of Oxygen Interstitial in Nb–V–O System

The distribution of an interstitial atom in a certain octahedral site $c_{u}^{O}$ mainly depends on the octahedral site energy $e_{u}^{O}$, and can be calculated by the well-known Fermi–Dirac partition function [22]:

(1) cuO=PuO1+exp[(euO−f(c))kT]

(*u* = 0, 1, …, 6)

where $P_{u}^{O}$ is the existing probability of different octahedral sites, f(c) represents the interstitial–interstitial atom interaction term and only depends on the total interstitial atoms’ concentration *C* with the boundary condition $C=\sum_{u=0}^{6}c_{u}^{O}$.

**Figure 3:**
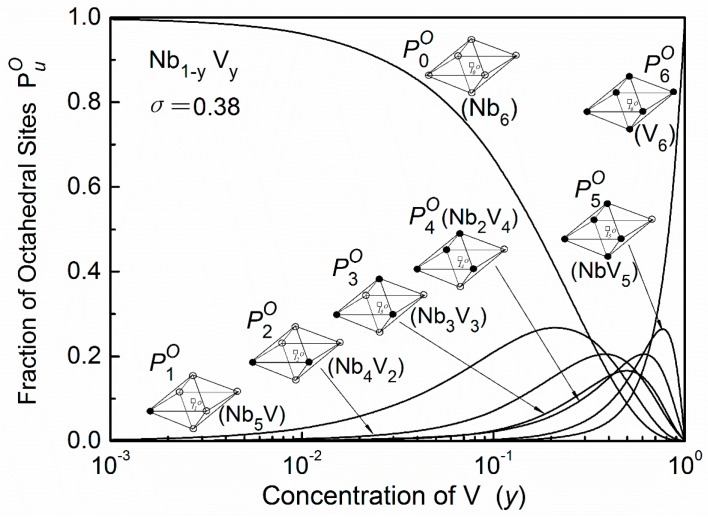
Fracion *P_u_^O^* of octahedral sites of a short range ordered Nb–V system (*σ* = 0.38). The sites are distinguished by the different chemical composition in the first shell of neighbors. (○—Nb atoms, ●—V atoms).

The probabilities of different octahedral sites $P_{u}^{O}$ are strongly influenced by the local order parameter (*σ*) of the system. It can be calculated by the following formula, which has been derived in Reference [4,18]:

(2) $$\left(P_{u}^{O}(\sigma )\right)=\left(\begin{array}{ccccc} P_{A}P_{AA} & 0 & 0 & 0 & 0\\ P_{A}P_{AB}+P_{B}P_{BA} & P_{A}P_{AA} & 0 & 0 & 0\\ P_{B}P_{BB} & P_{A}P_{AB}+P_{B}P_{BA} & P_{A}P_{AA} & 0 & 0\\ 0 & P_{B}P_{BB} & P_{A}P_{AB}+P_{B}P_{BA} & P_{A}P_{AA} & 0\\ 0 & 0 & P_{B}P_{BB} & P_{A}P_{AB}+P_{B}P_{BA} & P_{A}P_{AA}\\ 0 & 0 & 0 & P_{B}P_{BB} & P_{A}P_{AB}+P_{B}P_{BA}\\ 0 & 0 & 0 & 0 & P_{B}P_{BB} \end{array}\right)\left(P_{v}^{T}(\sigma )\right)$$

(*u* = 0, 1, …, 6, *v* = 0, 1, …, 4)

where *P_A_* or *P_B_* is the probability of the lattice atom A or B, *P*_*AA*(*B*)_ is the probability of finding an A (or B) atom as nearest neighbor of another A atom, $P_{u}^{T}$ is the probability of the tetrahedral site [5]. The calculation results of seven octahedral interstices $P_{u}^{O}$ in Nb_1−y_V_y_ system are shown in Figure 3. Here the local order parameter (*σ*) was chosen as 0.38 since this value was determined at a range of 0.33–0.44 by the enthalpy of solution data [19].

The site energy ($e_{u}^{O}$) of an interstitial atom mainly depends on the chemical affinity (Λ) between interstitial atoms and the nearest neighbor metal atoms, and on the elastic interaction of the local strain field (*ε*) of an octahedral cell in the matrix system [7]. It can be expressed:

(3) $$e_{u}^{O}=e_{u}^{O}(\Lambda )+e_{u}^{O}(\varepsilon ).$$

**Figure 4:**
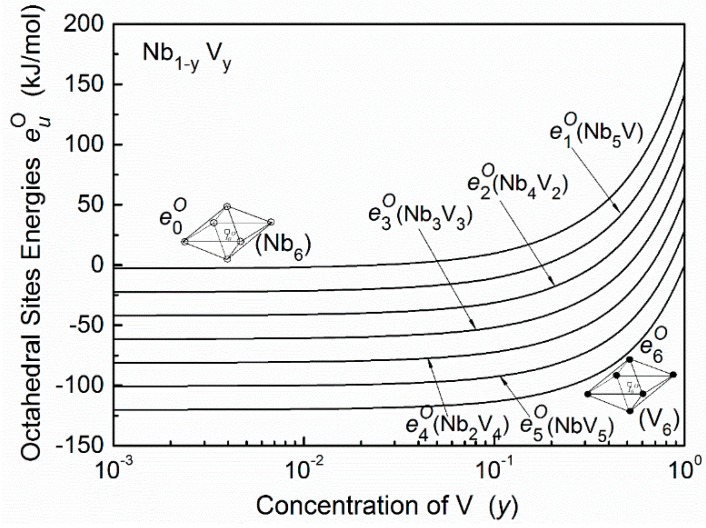
The octahedral site energy $e_{u}^{O}$ of oxygen atoms in Nb–V alloys as a function of alloy composition. (○—Nb atoms, ●—V atoms).

The first term $e_{u}^{O}(\Lambda )$ in Equation (3) can be approximately estimated by the difference of the formation enthalpy of a steady-state compound (interstitial-host) in the embedded octahedron cell and in the matrix alloys. The second term $e_{u}^{O}(\varepsilon )$ can be calculated by the different bulk modulus of the cell prior to and after embedding. The detailed procedures of the calculation of both terms were described in References [4] and [18]. The results of the octahedral site energy $e_{u}^{O}$ of the oxygen interstitial atoms in Nb–V alloys are shown in Figure 4. Here, NbO and VO are used in the calculation of $e_{u}^{O}(\Lambda )$ and $e_{u}^{O}(\varepsilon )$.

Then, by using Equations (1)–(3), the distribution of oxygen interstitial atoms in the Nb–V–O system can be determined under any thermal equilibrium conditions.

### 2.2. Dynamic Relaxation of the Interstitial Atoms

The Snoek relaxation results from the stress-induced transitions of interstitial atoms between the adjacent octahedral interstitial sites, i.e., $\sum_{u}I_{u}^{O}\to I_{u^{/}}^{O}$. As suggested in References [4,23], the saddle-point energy of such transition appears at the tetrahedral interstitial site $I_{v}^{T}$ between two adjacent octahedral sites. Thus, the saddle-point energy can be treated as the tetrahedral site energy $e_{v}^{T}$. Obviously, the activation energy $H_{m}{}_{u^{/}}$ of the transition can be calculated by the following expression.

(4) $$H_{uu^{/}}=e_{v}^{T}-e_{u}^{O}$$

(*u* = 0, 1, …, 6, *v* = 0, 1, …, 4)

**Figure 5:**
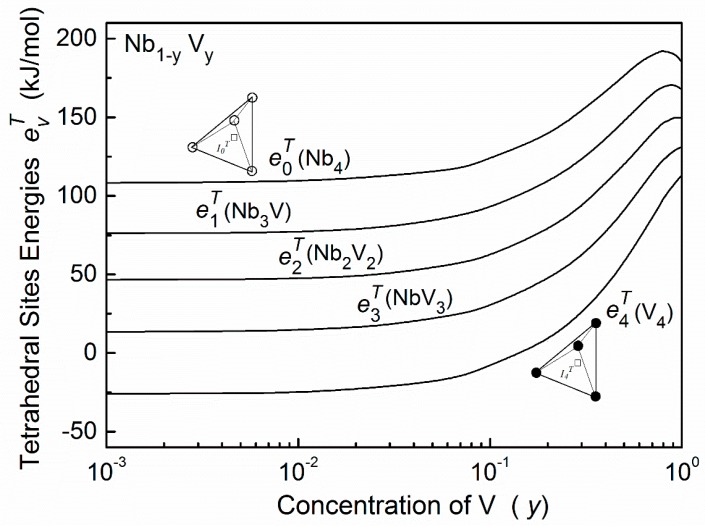
Tetrahedral site energy $e_{v}^{T}$ of oxygen atoms in Nb–V alloys. This energy is also treated as the saddle-point energy for oxygen atoms migrating. (○—Nb atoms, ●—V atoms).

The site energy $e_{v}^{T}$ can also be calculated by the embedded-cell method [4]. Here, the embedded cells are tetrahedrons, i.e., A*_4−v_*B*_v_* (*v* = 0, 1, …, 4). The calculated results of tetrahedral site energies $e_{v}^{T}$ in the Nb–V–O alloys are shown in Figure 5.

From the statistics point of view, the probability for occurrence of a transition $I_{u}^{O}\to I_{u^{/}}^{O}$ is proportional to a joint factor $\pi_{uu^{/}}^{O}$ as given by [24,25]:

(5) $$\pi_{uu^{/}}^{O}=c_{u}^{O}q_{uu^{/}}^{O}(1-\frac{c_{u^{/}}^{O}}{P_{u^{/}}^{O}})$$

where $1-\frac{c_{u^{/}}^{O}}{p_{u^{/}}^{O}}$ represents the conditional probability that, given an occupied site $I_{u}^{O}$, site $I_{u^{/}}^{O}$ is not occupied; and $q_{uu^{/}}^{O}$ is the conditional probability that, given an octahedral interstitial site of type *u*, another site selected among the sites adjacent to the first one is of type *u*^/^. $q_{uu^{/}}^{O}$ can be treated as a function about the matrix alloy composition [25].

The strength Δ of the overall Snoek relaxation can be expressed as the sum of terms corresponding to all possible transitions between the adjacent octahedral interstitial sites of the various types, i.e.,

(6) $$\Delta =\sum_{u}K_{uu^{/}}\pi_{uu^{/}}^{O}$$

where the coefficient $K_{uu^{/}}^{}$ represents the relaxation strength of a unit transition.

The temperature dependence of a Snoek relaxation, which contains several elementary Debye processes, can be described by the following expression [26]:

(7) $$Q^{-1}(T)=\sum_{u}K_{uu^{/}}\pi_{uu^{/}}^{O}\sec h[\frac{H_{uu^{/}}}{R}(\frac{1}{T}-\frac{1}{T_{m}{}_{uu^{/}}})]$$

where $T_{m}{}_{uu^{/}}$ is the peak temperature of the corresponding jump. Marx and Wert have shown a linear correlation between $H_{uu^{/}}$ and $T_{m}^{}{}_{uu^{/}}$ for Snoek-type peaks, i.e., the so-called Marx–Wert equation [27]:

(8) $$H_{uu^{/}}(T_{m}{}_{uu^{/}})=RT_{m}{}_{uu^{/}}\ln(\frac{BT_{m}^{}{}_{uu^{/}}}{hf_{m}})+T_{m}^{}{}_{uu^{/}}\Delta S$$

where *B* and *h* are the Boltzmann and Plank constants, ΔS is the entropy of the activation process, and *f*_m_ is the measuring frequency. For the entropy of activation, ΔS = 1.1 × 10^−4^ eV/K was usually used as it gave the best matching of single experiments with those carried out by the Arrhenius plot for the Debye relaxation [4,26].

From Equations (5)–(8), a Snoek relaxation peak in a ternary bcc system could be calculated in principle, and on the other hand, a complex anelastic experimental spectrum could be resolved into elementary relaxation processes based on the appropriate fitting procedure. These elementary processes should be the natural mechanism of the whole relaxation profile.

In the following section, this method will be applied to analyze the relaxation curves of the Nb–V–O alloys from the published investigations. In Section 3.1 and Section 3.2, we will consider the relaxation behaviors of Nb- and V-based Nb–V–O alloys with diluted substitutional solutes, respectively. In Section 3.3, we will briefly discuss the situation with concentrated substitutional solutes.

## 3. Results and Discussions

### 3.1. Results of Nb-Based Alloys

The compositional dependence of the Snoek relaxation curves in Nb-based Nb–V–O systems have been measured in detail in Carlson et al.’s work [6]. As an example, the results of Nb–0.5V–O (at. %) systems are chosen to reanalyze here.

**Figure 6:**
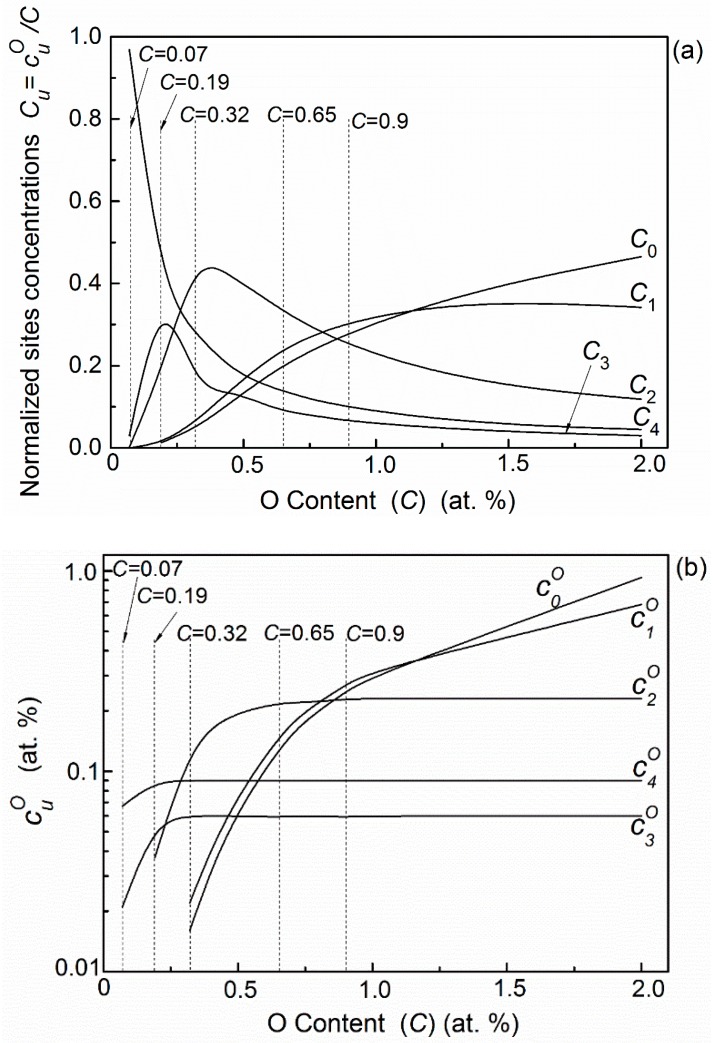
The distribution of oxygen atoms in Nb–0.5V (at. %) alloys with different oxygen content ($C=\sum_{u=0}^{6}c_{u}^{O}$): (**a**) normalized distributions *C_u_* ($C_{u}=c_{u}^{O}/C$); (**b**) absolute distribution $c_{u}^{O}$.

In this alloy the existing probabilities of octahedral sites $I_{5}^{O}$ and $I_{6}^{O}$ are quite low, thus oxygen atoms mainly distribute in the remaining five octahedral interstitial sites: $I_{u}^{O}$ (*u* = 0, 1, 2, 3, 4). The normalized ($C_{u}=c_{u}^{O}/C$) and absolute ($c_{u}^{O}$) distributions of the oxygen atoms in Nb–0.5V alloys are shown in Figure 6a,b, respectively. It can be seen that it also depends on the oxygen concentration. With the increase of oxygen content, oxygen atoms will preferentially occupy the octahedron sites of $I_{4}^{O}$, $I_{3}^{O}$, and then $I_{2}^{O}$, which contain four, three, and two nearest substitutional vanadium atoms respectively, due to more favorable site-dependent energies. When oxygen concentration increases further, the distribution of $c_{4}^{O}$, $c_{3}^{O}$, and $c_{2}^{O}$ will tend to saturation, and residual oxygen will, and have to, occupy the high-energy sites $I_{1}^{O}$ and $I_{o}^{O}$.

The conditional probabilities $q_{uu^{/}}^{O}$ of all possible transitions in this system are listed in Table 1. Although a large number of elementary relaxation processes are theoretically possible, some of them can be neglected due to their poor transition probabilities. Such kinds of transitions are highlighted in gray shadow in Table 1.

#### 3.1.1. In the Case of $\frac{C_{O}}{C_{V}}$ < 1

As shown in Figure 6, since the oxygen concentration is rather low in this case, the distribution status of oxygen atoms is rather simple. Therefore, only one experimental peak (i.e., P_II_) was observed in these systems.

**Figure 7:**
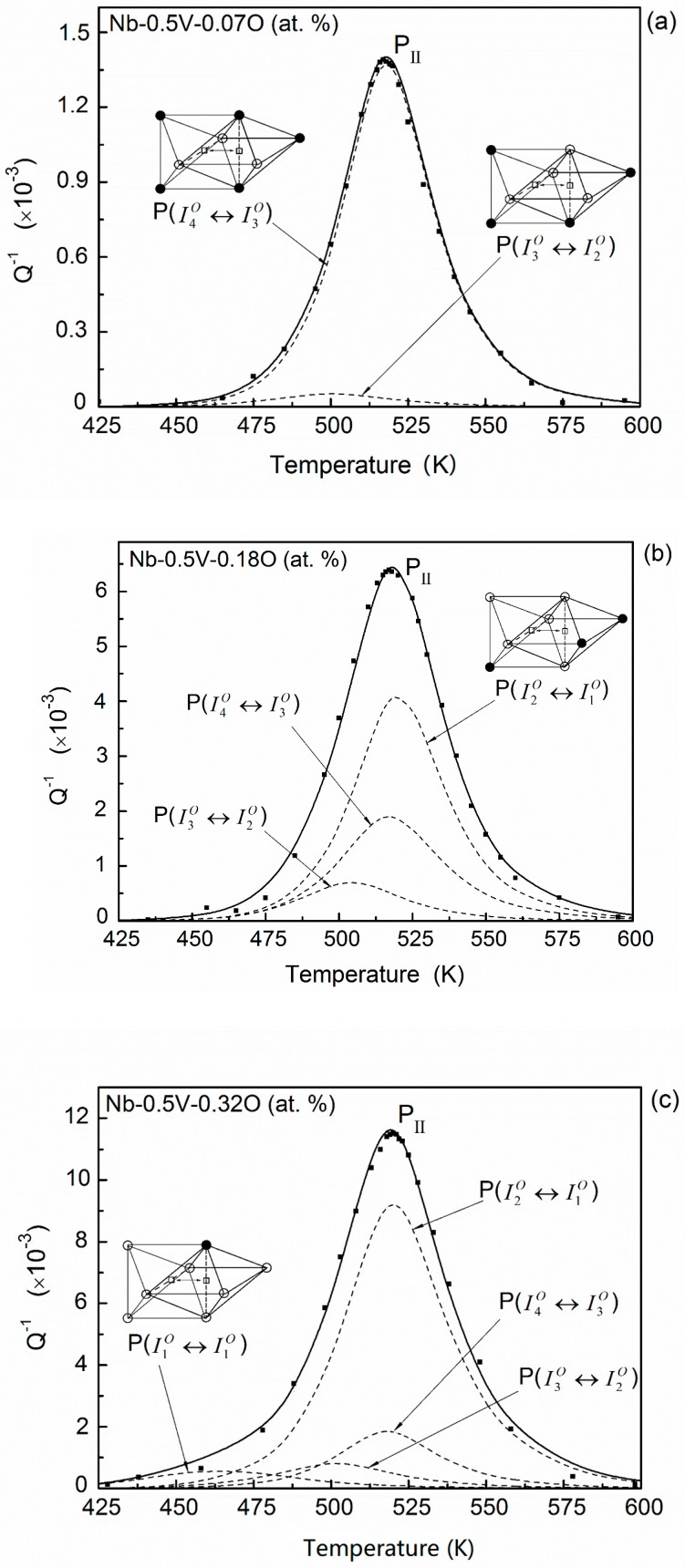
Temperature dependence of the relaxation peak and the fitting results for (**a**) Nb–0.5V–0.07O, (**b**) Nb–0.5V–0.18O, and (**c**) Nb–0.5V–0.32O (at. %) alloys (points—experimental; dashed line—fitted elementary peaks; solid line—the sum of the elementary peak). (○—Nb atoms, ●—V atoms).

In Nb–0.5V–0.07O (at. %) alloys, oxygen atoms mainly distribute in the sites $I_{4}^{O}$ and $I_{3}^{O}$. Based on the transition probabilities in Table 1, there are five possible types of transitions that could occur between adjacent interstitial sites: $I_{4}^{O}↔I_{2}^{O}$, $I_{4}^{O}↔I_{3}^{O}$, $I_{3}^{O}↔I_{1}^{O}$, $I_{3}^{O}↔I_{2}^{O}$, and $I_{3}^{O}↔I_{3}^{O}$. The spatial configurations and the relaxation parameters of these transitions are given in Table 2. Considering the transition probabilities, the main relaxation effects in this alloy should be due to the transitions of $I_{4}^{O}↔I_{3}^{O}$, $I_{4}^{O}↔I_{2}^{O}$, and $I_{3}^{O}↔I_{2}^{O}$. It needs to be noted that the transitions $I_{4}^{O}↔I_{3}^{O}$ and $I_{4}^{O}↔I_{2}^{O}$ have the same activation energy and peak temperature. Thus, they can be merged into one elementary process $I_{4}^{O}↔I_{3}^{O}$ as it has an absolute majority transition probability. By fitting the shape of the experimental results, two main elementary peaks can be determined. The fitting result is plotted in Figure 7a. The dashed lines represent the two relaxation processes proposed, and the full line corresponds to the sum of relaxation processes. The result shows that the fitting curve has an essentially good coincident with the peak (i.e., P_II_) encompassed by the experimental points.

The result of Nb–0.5V–0.18O (at. %) system is shown in Figure 7b. Besides occupying the interstitial sites of $I_{4}^{O}$ and $I_{3}^{O}$, about 20% of oxygen interstitial atoms will distribute in the site of $I_{2}^{O}$ in this alloy. Accordingly, new elementary relaxation processes resulted from the transitions of oxygen atoms in site $I_{2}^{O}$, will occur. Since the transition situation of the oxygen atoms in this system is more complicated than the case of Nb–0.5V–0.07O (at. %), the experimental relaxation peak P_II_ of Nb–0.5V–0.18O (at. %) alloys become broader than the former’s, as shown in Figure 1. Three main transitions, $I_{4}^{O}↔I_{3}^{O}$, $I_{3}^{O}↔I_{2}^{O}$, and $I_{2}^{O}↔I_{1}^{O}$, can be identified in this system. Although in this case, most of oxygen atoms distribute in the site of $I_{4}^{O}$ and the main transitions of oxygen atoms occur in the process $I_{4}^{O}↔I_{3}^{O}$, the most effective relaxation process is due to transition of $I_{2}^{O}↔I_{1}^{O}$. The reason should be that the strength of the Snoek relaxation depends not only on the interstitial solute concentration of the transition, but also on the strain field around the interstitial solute atom and on the symmetry characteristics of the point-defect reorientation responses [28].

In Nb–0.5V–0.32O (at. %) alloys, the concentrations of oxygen atoms in the sites of $I_{4}^{O}$ and $I_{3}^{O}$, i.e., $c_{4}^{O}$ and $c_{3}^{O}$, become saturated, and more than 40% of oxygen atoms distribute in the site of $I_{2}^{O}$. Additionally, the occupancies of the higher-energy site $I_{1}^{O}$ become appreciable and the corresponding transition $I_{1}^{O}↔I_{1}^{O}$ may also contribute to the whole relaxation performance. This additional process will further increase the width of whole relaxation peak (P_II_) in this system. The fitting result is shown in Figure 7c.

#### 3.1.2. In the Case of $\frac{C_{O}}{C_{V}}$ > 1

Now we consider the relaxation performance for the Nb–0.5V (at. %) alloys which contain a high oxygen concentration.

**Figure 8:**
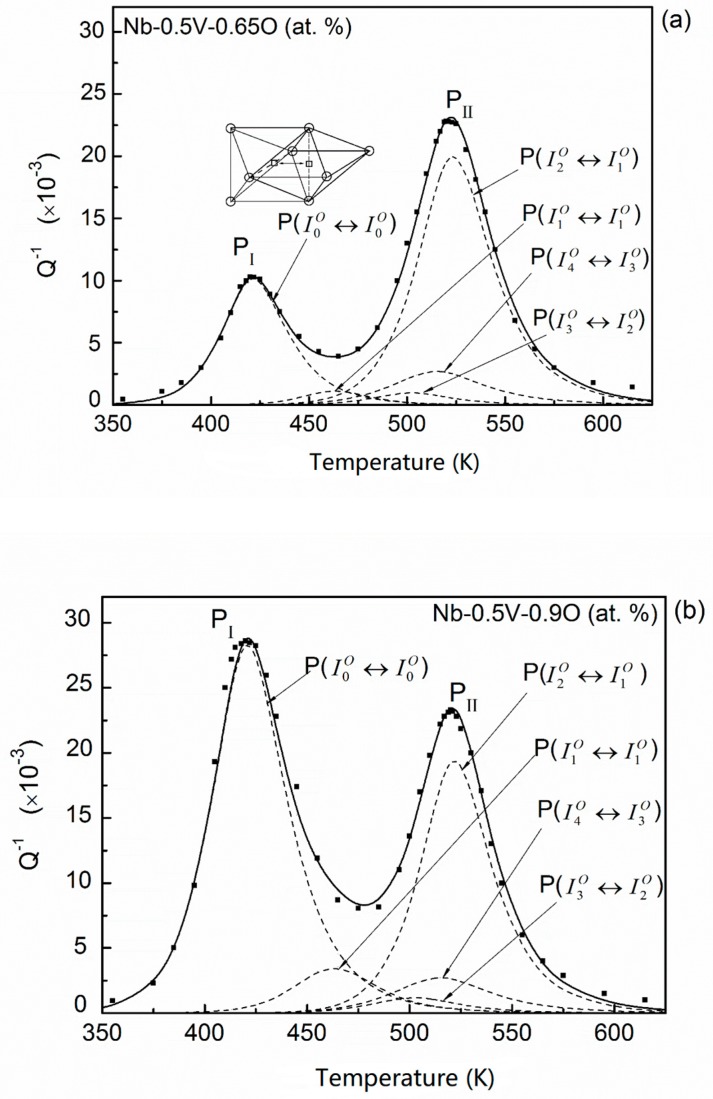
Temperature dependence of the relaxation peak and the fitting results for (**a**) Nb–0.5V–0.65O, and (**b**) Nb–0.5V–0.9O (at. %) alloys (points—experimental; dashed line—fitted elementary peaks; solid line—the sum of the elementary peak). (○—Nb atoms, ●—V atoms).

As shown in Figure 6, with the increase in oxygen concentration, the occupying of energy-favorable sites such as $I_{4}^{O}$, $I_{3}^{O}$, and $I_{2}^{O}$ will reach the saturated capacities. These situations are fully exhibited in the case of the Nb–0.5V–0.65O (at. %) system. In addition, about 23.6% and 19.8% of oxygen atoms distribute in the sites of $I_{1}^{O}$ and $I_{0}^{O}$, respectively. Almost all transitions of oxygen interstitial atoms in $I_{0}^{O}$ site occur in the process of $I_{0}^{O}↔I_{0}^{O}$ since the transition probability of $q_{00^{/}}^{O}$ is equal to 0.99. This process is the so-called normal oxygen Snoek relaxation in unalloyed Nb, which has been reported elsewhere at about 420 K (at frequency 1 Hz). Thus, a second peak (P_I_) at about 400 K was observed in the experimental curve. The fitting results are plotted in Figure 8a. It can be seen that the experimental peak P_I_ mainly resulted from the elementary relaxation process $I_{0}^{O}↔I_{0}^{O}$. The height of fitted elementary peaks P($I_{4}^{O}↔I_{3}^{O}$) and P($I_{3}^{O}↔I_{2}^{O}$) in this alloy are nearly equal to the case of the Nb–0.5V–0.32O alloy for the same distributions of $c_{4}^{O}$ and $c_{3}^{O}$ in both systems.

The experimental results for Nb–0.5V–0.9O (at. %) is similar to the case of Nb–0.5V–0.65O (at. %) but the height of peak P_I_. The fitting results are shown in Figure 8b. In this alloy, most of the oxygen atoms distribute in the site of $I_{1}^{O}$ and $I_{0}^{O}$, thus the peak P_I_ is much higher than that of the Nb–0.5V–0.65O alloy.

Based on above analysis, we can clarify the origin of the experimental peak P_II_ in Carlson et al.’s investigations [6]. It can be seen that peak P_II_ mainly comprises of three elementary relaxation processes: P($I_{4}^{O}↔I_{3}^{O}$), P($I_{3}^{O}↔I_{2}^{O}$), and P($I_{2}^{O}↔I_{1}^{O}$). Thus, this peak strongly depends on the oxygen distributions in the sites of $I_{4}^{O}$, $I_{3}^{O}$, and $I_{2}^{O}$. As previously mentioned, when the oxygen concentration exceeds a certain level, the occupying of these sites will tend to the saturated capacities, consequently, this peak will reach the saturated height. This is the reason why the peak P_II_ for the Nb–0.5V–0.65O (at. %) and Nb–0.5V–0.9O (at. %) systems has the same maximum height and the same peak temperature.

**Figure 9:**
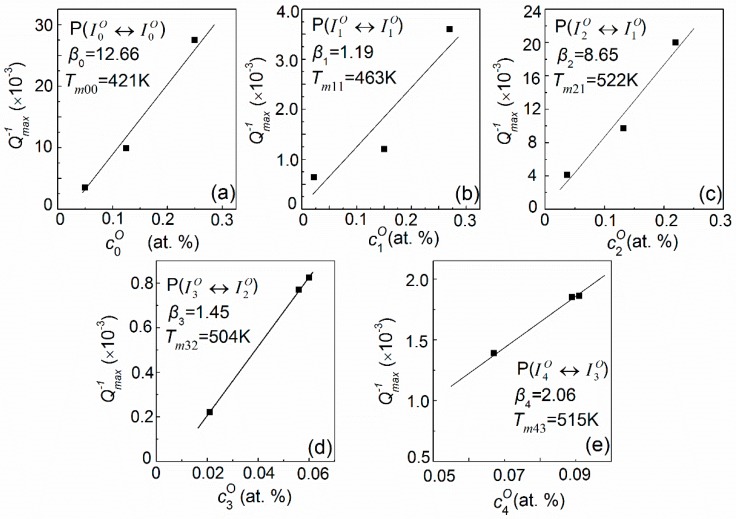
Plots of *Q_u_*^−1^*_m_* vs. $c_{u}^{O}$ for the fitted elementary peaks in the Nb–0.5V (at. %) alloys: (**a**) P($I_{0}^{O}↔I_{0}^{O}$); (**b**) P($I_{1}^{O}↔I_{1}^{O}$); (**c**) P($I_{2}^{O}↔I_{1}^{O}$); (**d**) P($I_{3}^{O}↔I_{2}^{O}$); (**e**) P($I_{4}^{O}↔I_{3}^{O}$).

**Figure 10:**
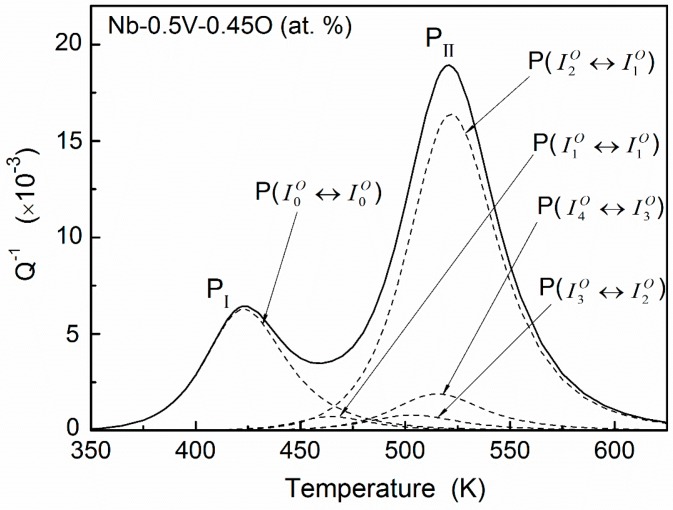
The calculated relaxation curve for the Nb–0.5V–0.45O (at. %) system. A notable peak P_I_ can be determined in this system although the oxygen concentration is lower than that of vanadium solutes.

The heights of all fitted elementary peaks (*Q_u_*^−1^*_m_*) for Nb–0.5V (at. %) alloys as a function of oxygen site concentration ($c_{u}^{O}$) are plotted in Figure 9. It can be seen that, basically, a good linear relation exists between *Q_u_*^−1^*_m_* and $c_{u}^{O}$, i.e., *Q_u_*^−1^*_m_* = *β*$c_{u}^{O}$. This is also a characteristic of the standard Snoek-type relaxation process [1]. Using these linear relations and the distributions of the oxygen solutes, as shown in Figure 6, the temperature-dependence relaxation curve for Nb–0.5V systems with any oxygen concentration can be determined in principle. As a matter of example, the calculated curve for Nb–0.5V–0.45O (at. %) is shown in Figure 10. It can be seen that, although $\frac{C_{O}}{C_{V}}$ < 1 in this system, a notable peak P_I_ also can be predicted, rather than what Carlson et al. proposed. Such a characteristic mainly results from the appreciable distribution (about 10.94% as shown in Figure 7a) of oxygen atoms in the interstitial sites of $I_{0}^{O}$.

### 3.2. Results of V-Based Alloys

As mentioned in the introduction, the relaxation peak (P_III_) width of V-based Nb–V–O alloys is not broadened with an increase in the oxygen content. It indicates that the distribution and transition situations of the oxygen atoms in a V-rich system are insensitive to the oxygen concentrations. Now we reanalyze these results based on the present method.

**Figure 11:**
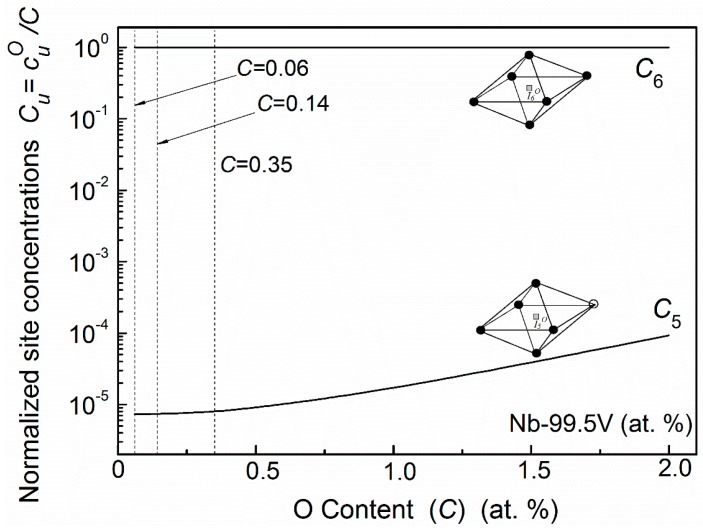
Normalized distribution of oxygen atom in Nb–99.5V (at. %) alloys with different oxygen content. (○—Nb atoms, ●—V atoms).

**Figure 12:**
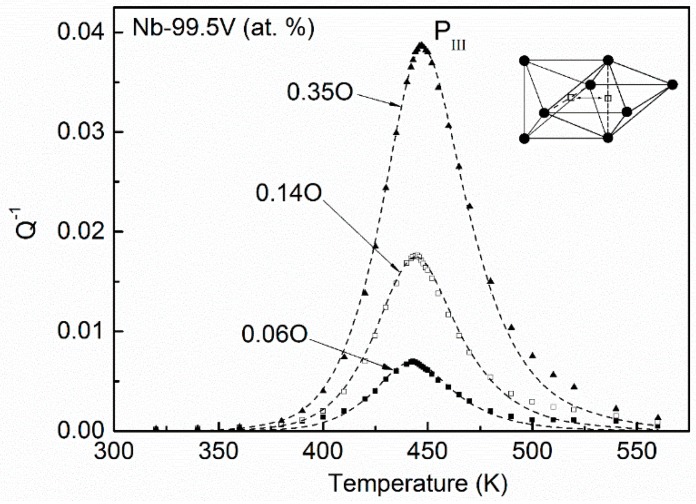
Temperature dependence of the relaxation peak and the fitting results for Nb–99.5V alloys with 0.06, 0.14, and 0.35 at. % O (points—experimental; dashed line—fitted elementary peaks using a single Debye process). (○—Nb atoms, ●—V atoms).

The distribution statuses of oxygen atoms for Nb–99.5V (at. %) alloys with different oxygen content are shown in Figure 11. Almost all oxygen atoms in this system distribute in the octahedral site of $I_{6}^{O}$ which has six nearest vanadium substitutional solutes. Only with a rather high oxygen concentration will a minute quantity of oxygen atoms occupy the $I_{5}^{O}$ site. Therefore, the main relaxation transition in V-based Nb–VO alloys should occur in the process of $I_{6}^{O}↔I_{6}^{O}$, and the influence of oxygen atoms in $I_{5}^{O}$ site can be neglected. The fitting results for Nb–99.5V (at. %) alloys with three kinds of oxygen concentrations are plotted in Figure 12. One can see that the experimental curve can be successfully resolved by using a single Debye peak, i.e., P($I_{6}^{O}↔I_{6}^{O}$). The relaxation mechanism in a V-based Nb–V–O system is rather simple compared with the situation in an Nb-based system.

From the above, we can see that the experimental peak P_III_ in V-based alloys is, indeed, an oxygen Snoek peak in unalloyed vanadium. It can be expected that, even if the oxygen concentration exceeds substitutional niobium content (i.e., $\frac{C_{O}}{C_{Nb}}$ > 1), no another relaxation peak would be observed in this system for a simple distribution status of the oxygen atoms.

Although this peak is not perturbed by substitutional niobium solutes in a V-based alloy, there is no trace of evidence for antitrapping of oxygen by niobium atoms as proposed by Carlson et al. [6]. In fact, such a viewpoint was controversial to the kinetic measured results of oxygen in Nb–V alloys by Park and Altstetter [17]. The diffusivity measurement has shown that vanadium decreases the diffusion coefficient of oxygen significantly in Nb-rich alloys, whereas niobium has a similar effect, but to a smaller degree, in V-rich systems. It could be seen that niobium also has trapping effects to oxygen atoms in vanadium, of course, weaker than the vanadium atom does in Nb-rich alloys.

### 3.3. Results of Concentrated Substitutional Solutions Alloys

**Figure 13:**
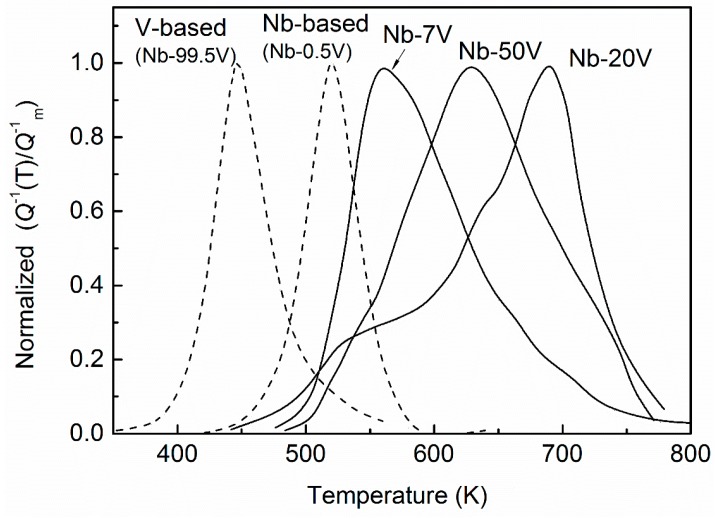
Normalized relaxation peaks for concentrated substitutional solute Nb–V alloys with 7, 20, and 50 at. % V. Comparison with the peaks for Nb-based (Nb–0.5V (at. %)) and V-based (Nb–99.5V (at. %)) systems.

The relaxation performances for Nb–V–O alloys with concentrated substitutional vanadium solutes were investigated in References [10,12,13]. As shown in Figure 13, with increasing vanadium concentrations the spectra present notable broadening and asymmetry, and significantly shift to a higher temperature, and for a given oxygen concentration the peak value decreases when the substitutional vanadium content is increased. In these works, the broadened effect of the peaks were simply attributed to the reorientation of the oxygen atoms associated with two or more nearest substitutional solutes in one octahedral cell. However, as admitted in Reference [12], “the reality should be much more complicated” since a Snoek transition of interstitial atoms must occur between two adjacent octahedral sites.

In principle, based on the present statistical treatment, the relaxation curve for these kinds of Nb–V–O systems can also be resolved into a series of identifiable elementary peaks if these elementary processes can be identified case by case. However, with increasing substitutional solute concentration, the distribution statuses of the oxygen atoms become quite complicated since all seven types of octahedral sites have similar levels of the exiting probabilities, as shown in Figure 3. In addition, the transition probabilities of all possible elementary processes in concentrated substitutional systems become appreciable. For example, in the case of the Nb–50V (at. %) system, all 29 kinds of transitions have considerable probabilities, as shown in Table 3, which means that all possible elementary processes in this system cannot be neglected. Consequently, it is imaginable that a large number of oxygen atom transitions could occur for relaxations in such kinds of systems.

In fact, if the symmetry features associated with the distribution of substitutional solutes and the multiform spatial configurations between the adjacent octahedron are also taken into account [29], many more kinds of elementary relaxation processes can be expected in Nb–7V, Nb–20V, and Nb–50V (at. %) alloys, respectively. As a consequence, it may not be possible to accurately resolve one relaxation process from another and in some extreme cases they may only contribute to an increase of background dissipation. Of course, relative strength of the various elementary processes may be considerably different and some of them may be undetectable experimentally. Thus, we can understand qualitatively that with increasing substitutional vanadium concentration the relaxation peak width becomes larger, and the peak height decreases. This is not a unique relaxation characteristic only for Nb–V–O alloys with concentrated substitutional solutes as similar results are also observed in other ternary systems, such as Nb–Ti–O [30], Ni–Fe(Pd)–H(D) [31] and Ta–Re–O(N) [32] etc.

## 4. Conclusions

The Snoek relaxation peaks for the ternary bcc Nb–V–O systems, which have been reported in the published investigations, are reanalyzed by using an extended statistical mechanical method. The relaxation characteristics and mechanism are discussed and clarified.

(1) The complicated Snoek spectrums of a Nb–V–O system can be, in principle, resolved into several effective elementary Debye peaks, which correspond to the transitions of interstitial oxygen atoms between adjacent octahedral sites. The relaxation parameters of each elementary peak can be determined by element species and atomic arrangements within the corresponding octahedron.

(2) The Snoek relaxation characteristic in Nb–V–O systems mainly depends on the site distributions and the transitions status of the interstitial oxygen atoms.

(3) The relaxation peak (P_II_) presented at about 520 K in Nb-based Nb–V–O alloys is not a simple V–O interaction peak. This peak is mainly due to the oxygen atom transitions in three kinds of octahedral sites, which have four, three, and two nearest vanadium substitutional atoms. Consequently, it comprises at least three elementary relaxation processes. On the other hand, in a V-based Nb–V–O system, interstitial oxygen atoms mainly distribute in the octahedron composed by six vanadium atoms. Therefore, the peak (P_III_) presented at about 440 K in this system is an unperturbed oxygen Snoek peak in vanadium.

(4) In the case of Nb–V–O systems with concentrated substitutional vanadium solutes, the notable broadening effect of the relaxation spectrum mainly results from the multiform distribution status and numerous possible elementary transitions of the oxygen atoms.

## Figures and Tables

**Table 1:** Conditional probabilities $q_{uu}^{O}$ of all possible transition in Nb-0.5V alloys.

$q_{uu}^{O}$	$I_{0}^{O}$	$I_{1}^{O}$	$I_{2}^{O}$	$I_{3}^{O}$	$I_{4}^{O}$	$I_{5}^{O}$	$I_{6}^{O}$
$I_{0}^{O}$	0.99	1.00 × 10^−2^	2.50 × 10^−5^				
$I_{1}^{O}$	0.207	0.785	7.50 × 10^−3^	1.88 × 10^−5^			
$I_{2}^{O}$	6.19 × 10^−2^	0.596	0.338	4.40 × 10^−3^	1.09 × 10^−5^		
$I_{3}^{O}$		0.136	0.721	0.141	1.90 × 10^−3^	4.68 × 10^−6^	
$I_{4}^{O}$			0.333	0.599	6.69 × 10^−2^	6.34 × 10^−4^	1.56 × 10^−6^

**Table 2:** The configurations and the relaxation parameters of all possible transitions of oxygen solute atoms in Nb–0.5V–0.07O (at. %) alloys (○—Nb atoms, ●—V atoms).

Transition Type	Configuration	Transition Probability πuu/O∑uπuu/O (Normalized)	Site-Energy $e_{u}^{O}$ (kJ/mol)	Saddle-Point Energy $e_{v}^{T}$ (kJ/mol)	Activation Energy $H_{u}^{}{}_{u^{/}}^{}$ (kJ/mol)	Peak Temperature $T_{m}^{}{}_{uu^{/}}$ (K)
$I_{4}^{O}↔I_{2}^{O}$	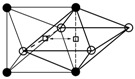	0.146	−77.79	51.35	129.14	515
$I_{4}^{O}↔I_{3}^{O}$	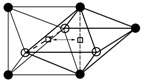	0.822	−77.79	51.35	129.14	515
$I_{3}^{O}↔I_{1}^{O}$	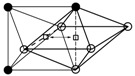	4.3 × 10^−3^	−58.19	69.60	127.79	504
$I_{3}^{O}↔I_{2}^{O}$	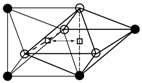	0.0229	−58.19	69.60	127.79	504
$I_{3}^{O}↔I_{3}^{O}$	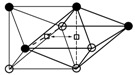	4.5 × 10^−3^	−58.19	51.35	109.54	440

**Table 3:** Conditional probabilities $q_{uu^{/}}^{O}$ of all possible transitions in Nb–50V alloys.

$q_{uu^{/}}^{O}$	$I_{o^{/}}^{O}$	$I_{1^{/}}^{O}$	$I_{2^{/}}^{O}$	$I_{3^{/}}^{O}$	$I_{4^{/}}^{O}$	$I_{5^{/}}^{O}$	$I_{6^{/}}^{O}$
$I_{o}^{O}$	0.25	0.50	0.25				
$I_{1}^{O}$	0.0625	0.3125	0.4375	0.1875			
$I_{2}^{O}$	0.0156	0.1563	0.3750	0.3438	0.1094		
$I_{3}^{O}$		0.0469	0.25	0.4063	0.25	0.0469	
$I_{4}^{O}$			0.1094	0.3438	0.3750	0.1563	0.0156
$I_{5}^{O}$				0.1875	0.4375	0.3125	0.0625
$I_{6}^{O}$					0.25	0.50	0.25
